# Selenium deficiency is linearly associated with hypoglycemia in healthy adults

**DOI:** 10.1016/j.redox.2020.101709

**Published:** 2020-09-01

**Authors:** Yue Wang, Eddy Rijntjes, Qian Wu, Hongjun Lv, Chuqi Gao, Bingyin Shi, Lutz Schomburg

**Affiliations:** a Department of Endocrinology, The First Affiliated Hospital of Xi'an Jiaotong University, Xi'an, 710061, China; b Institute for Experimental Endocrinology, Charité-Universitätsmedizin Berlin, CVK, Corporate Member of Freie Universität Berlin, Humboldt-Universität zu Berlin, And Berlin Institute of Health, 13353, Berlin, Germany; c Xi'an Jiaotong University Health Science Center, Xi'an, 710 061, China

**Keywords:** Selenium, Selenoprotein, SELENOP, GPX, Insulin, Redox

## Abstract

**Objective:**

The trace element selenium (Se) is needed for regular biosynthesis of selenoproteins, which contribute to antioxidative defense systems and affect redox-regulated signaling. Elevated Se intake and selenoprotein expression levels have been associated with impaired hydrogen peroxide-dependent signaling by insulin, leading to hyperglycemia and insulin resistance. The relation of low Se intake with glucose status and carbohydrate metabolism is poorly known.

**Research design and methods:**

A cross sectional analysis among healthy subjects residing in two Chinese counties with different habitual Se intakes was conducted. Fasted glucose levels were related to Se concentrations of 5686 adults by linear regression analysis with Se, body mass index, age, thyroid status, insulin and sex as independent variables.

**Results:**

Serum Se correlated strongly and positively with glucose in the Se-deficient population. There was no strong relationship of Se and glucose in the non-deficient population. Overt hypoglycemia (serum glucose < 2.8 mM) was observed in 19.2% of this random sample of subjects in the Se-deficient and in 1.4% of the moderately supplied population, respectively.

**Conclusions:**

An adequate Se supply constitutes an important factor for glucose homeostasis in human subjects. The interaction between Se status and glucose control is not limited to hyperglycemia, but apparently extends to hypoglycemia risk in Se deficiency. This newly identified relationship may be of relevance for the course of severe disease including major trauma, sepsis and COVID-19, where Se deficiency has been associated with mortality risk.

## Highlights

• A cross-sectional study compared serum Se and Glc levels in 5686 healthy adults.
• Half of the subjects were residing on Se poor soil and displayed Se deficiency.
• These Se-deficient subjects showed a linear association of serum Se and Glc.
• A well-matched control population with adequate Se supply showed no such relation.
• This novel interaction may be relevant for disease and death risk in Se deficiency.

## 1 Introduction

The association between selenium (Se) status and glucose metabolism, insulin resistance and type 2 diabetes risk is controversially discussed and poorly understood [1,2]. Cell culture studies have indicated that supplemental Se attenuates insulin signaling and glucose uptake in myotubes [3] and hepatocytes [4], respectively. In transgenic mice, global overexpression of the Se-dependent cytosolic glutathione peroxidase (GPx 1) leads to obesity and insulin resistance [5], whereas cell-type restricted overexpression in pancreatic beta cells specifically results in elevated insulin biosynthesis and secretion [6]. Along this line, artificially increasing the Se status above physiological levels by injection of Se-rich selenoprotein P is capable of directly inducing hyperglycemia and insulin resistance in mice [7]. It is unknown at present whether the endogenous control mechanisms limiting selenoprotein expression to a certain maximal value serve as an inborn barrier to protect the organism and especially the pancreas and insulin-target sites from surplus Se [1,2,8,9]. However, intervention studies have indicated that these maximal levels are no absolute limit, and high dosage Se application is capable of promoting selenoprotein concentrations above accepted limits [10].

In humans, serum Se levels seem to be unrelated to newly diagnosed type 2 diabetes [11], but are increased in patients with manifest type 2 diabetes [12]. Supplemental Se may increase type 2 diabetes risk in older subjects [13], specifically in males with high baseline Se status [14], but not in the general population [15]. Collectively, the different lines of research conducted in cell culture, animal models and humans have highlighted that the regular control of insulin biosynthesis, secretion and signaling depends on redox balance and the activity of selenoenzymes [16,17]. A strongly elevated Se intake increases selenoprotein expression and may directly impair redox signaling, beta cell function and insulin target cell response [17,18]. However, a recent meta-analysis of high quality randomized controlled trials indicated that the risk for type 2 diabetes mellitus is not affected by supplemental Se in a dosage of 200 μg/day provided as selenized yeast or l-selenomethionine [19].

While most of the clinical studies currently available are mainly concerned with Se-replete subjects and the potential risk of Se over-supplementation, the importance of an insufficient Se intake and habitual selenoprotein deficit for carbohydrate metabolism and euglycemia is unknown.

## 2 Research design and methods

A cross-sectional study of healthy subjects has been conducted in two regions in Shaanxi Province, China, i.e., Ziyang and Ningshan [20]. The study protocol had been approved by the Medical Ethics Committee of Xi'an Jiaotong University, China. Both groups had similar demographics including a comparable male:female ratio, occupation and smoking rates, and similar food-consumption patterns, except for a slight difference in alcohol intake [20]. The two regions, however, differ strongly in soil Se levels, resulting in an almost twofold lower serum Se status in subjects from Ningshan (low Se status) as compared to Ziyang (moderate Se status) [20]. The Se status in Ningshan is on average below the threshold needed for adequate selenoprotein expression, i.e., below total serum Se concentrations of 1.0–1.2 μM (79–95 μg/l) [21]. Concentrations at or below this threshold are generally considered as indicating Se deficiency [22,23].

A total of 3279 subjects were enrolled in Ziyang and 3373 in Ningshan, of whom 430 and 437, respectively, were excluded for incomplete data or insufficient serum samples, and 52 and 47, respectively, for being outliers in one of the parameters of interest (three times the Inter Quartile Range above the 75th or below the 25th percentile). Serum Se, TSH and thyroid hormones were determined as described [20]. Glucose and insulin concentrations were measured in venous blood samples collected after a ten-hour overnight fast. Assays were conducted on an automatic analyzer (LABOSPECT 008, Hitachi, Tokyo, Japan), using an enzymatic glucose assay (FUJIFILM Wako Pure Chemical Corporation, Japan). Intra- and inter-assay coefficients of variation were below 1%, determined with two standards of 5.3 and 11.9 mM glucose, respectively. To allow for regression analysis, insulin was determined with an extended standard curve for some of the sera.

*Statistical Analysis:* The two regions were analyzed separately. If applicable, data were normalized using an ln-transformation. A linear regression model was created for ln (glucose) using ln (Se), ln (BMI), age and sex as independent variables (model 1). A further model was created, using ln (insulin) in addition to the first model (model 2). As the thyroid status had no further contribution to the models (standardized beta for T3, T4 and TSH (-0.028 – 0.014); p > 0.1), it was excluded from the final models. P values < 0.05 were considered statistically significant. Data are presented as median and [inter quartile range].

## 3 Results

The population enrolled in Ziyang was slightly older (Ziyang: 50 [39–59] years; Ningshan: 47 [39–56] years), had a slightly lower BMI (Ziyang: 22.3 [20.5–24.4] kg/m^2^; Ningshan: 23.0 [21.0–25.4] kg/m^2^) and a considerably higher Se status (serum Se: Ziyang: 103 [79–134] μg/l; Ningshan: 58 [40–82] μg/l). Median glucose concentrations were significantly higher in Ziyang (5.19 [4.49–6.07] mmol/l) than in Ningshan (4.00 [3.02–5.53] mmol/l) (Table 1). Insulin concentrations were similar (Ziyang: 10.0 [7.0–20.5] mIUl/l; Ningshan: 9.4 [7.0–15.9] mIU/l); 40% and 38%, respectively, of the population had an insulin concentration below 7.0 mIU/l. In linear regression model 1, subjects from Ziyang showed a marginal relationship between serum Se and glucose levels (standardized beta 0.045, p = 0.018; Table 2). Gender did not contribute significantly to the linear regression models of glucose in either region (standardized beta 0.008–0.021; p > 0.1). There was a small effect of age (Ziyang: standardized beta 0.086, p < 0.001; Ningshan: standardized beta 0.112, p < 0.001) and BMI (Ziyang: standardized beta 0.031, p = 0.095; Ningshan: standardized beta 0.031, p = 0.034) (Table 2).

**Table 1:** Clinical characteristics of the subjects.

	Ziyang (n = 2797)*	Ningshan (n = 2889)*
	Median [IQR]	Median [IQR]
Glucose (mmol/l)	5.19 [4.49–6.07]	4.00 [3.02–5.53]
Insulin (mIU/l)	10.0 [7.0–20.5]	9.4 [7.0–15.9]
Selenium (μg/l)	103 [79–134]	58 [40–82]
BMI (kg/m^2^)	22.3 [20.5–24.4]	23.0 [21.0–25.4]
Age (y)	50 [39–59]	47 [39–56]
Gender (%m)	30.7%	32.2%

**Table 2:** Linear regression model showing the relationship between ln (glucose) and ln (Se) in Ziyang and Ningshan.

	Ziyang (n = 2797)					Ningshan (n = 2889)				
	Unstandardized coefficients		Stand. coefficient			Unstandardized coefficients		Stand. coefficient		
	B	Std Error	Beta	T	p-value	B	Std Error	Beta	T	p-value
**Constant**^a^	1.193	0.144	–	8.266	<0.001	-1.271	0.156	–	-8.135	<0.001
**Se (μg/l)**	0.030	0.013	0.045	2.371	0.018	0.529	0.012	0.619	42.718	<0.001
**BMI (kg/m**^**2**^**)**	0.070	0.042	0.031	1.671	0.095	0.101	0.047	0.031	2.124	0.034
**Age (y)**	0.002	0.000	0.086	4.522	<0.001	0.004	0.001	0.112	7.627	<0.001
**Gender (%male)**	0.005	0.011	0.008	0.419	0.675	0.019	0.013	0.021	1.432	0.152

a The linear regression model for ln (glucose) contained the ln (selenium), ln (BMI), age and gender as independent variables.

**Fig. 1:**
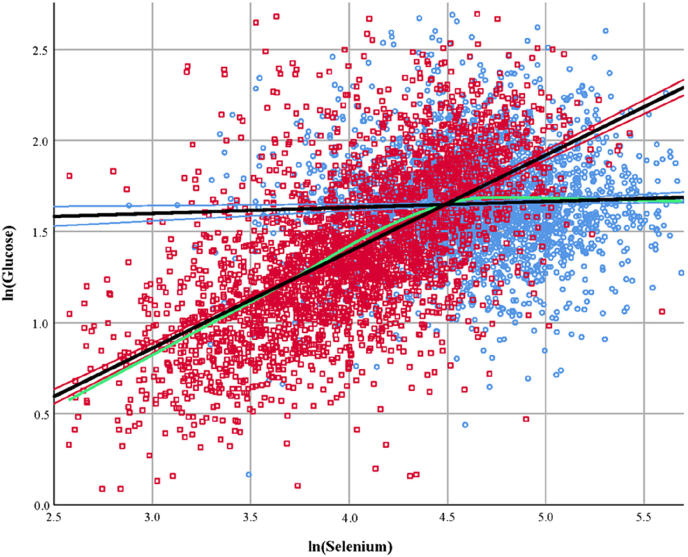
Scatterplot showing the relationship between ln (Se) and ln (Glucose) in the group of subjects residing in Ziyang (moderate Se status, blue circles) and Ningshan (low Se status, red squares). The black regression lines are based on linear regression and surrounded by the 95%-confidence interval. The lines intersect at a serum Se concentration of 88.9 μg/l (Ziyang: y = 1.501 + 0.033x; Ningshan: y = -0.7307 + 0.53x). The green line represents the locally estimated scatterplot smoothing curve (α = 50%) of the combined regions. (For interpretation of the references to colour in this figure legend, the reader is referred to the Web version of this article.)

A strong relationship between glucose and Se concentrations was found in the group of subjects from Ningshan (standardized beta 0.619, p < 0.001). The deduced model indicates that a change in ln (Se) by one standard deviation will result in a substantial 0.619 standard deviation change in ln (glucose) (Table 2). The differential relationship between glucose and Se concentrations for the two populations is emphasized by the green line in the XY-scatter plot (Fig. 1). This line represents a locally estimated scatterplot smoothing (LOESS) curve of the combined regions which is independent of the data distribution. The flattening of the LOESS curve after the linear regression lines intersect (Se at ~89 μg/l) indicates the relationship between Se and glucose is not strong, or maybe absent, in the moderately supplied subjects, but strong and pronounced in the subjects with low Se status. Addition of insulin to the model (Model 2) reduced the effect of the BMI on glucose concentrations, but further increased the effect of Se in the Ningshan region (standardized beta 0.733, p < 0.001).

## 4 Discussion and conclusions

The comparison of these two populations highlights a relationship of serum Se and glucose concentrations in Se-deficient subjects, which is not observed at serum Se levels above 89 μg/l. This finding identifies a hitherto unknown nutritional factor of high relevance for the risk of hypoglycemia. A certain minimal Se intake that is equivalent to the amount needed for full expression of the selenoenzyme glutathione peroxidase-3 (GPX3) [21], or an almost full expression of the Se transporter selenoprotein P (SELENOP) [24] seems to be required for achieving and maintaining euglycemia. Until now, inadequate diabetes medication, certain endocrinopathies, cancer or critical illness are among the few established causes of hypoglycemia [25]. The identification of Se deficiency as another hypoglycemia-associated condition provides a meaningful addition to the list of risk factors.

It is unlikely that the relationship observed is due to variations in energy intake, genetics, life style or other confounders, as both populations are highly similar and separated by a geographical distance of ca. 500 km only [20]. A moderate regular Se supply seems thus essential for achieving and maintaining euglycemia, likely due to allow sufficiently high expression of critical selenoproteins like intracellular GPX1 involved in redox signaling controlling insulin biosynthesis and insulin receptor activity [8,17,18]. Importantly, a deficit of Se and insufficient expression of intracellular selenoproteins would constitute a preventable and treatable condition that can easily be avoided by nutritional adaptations or supplemental measures [9].

During the collection of study samples, no obvious signs of hypoglycemia were noted in the subjects from Ningshan. In order to address this issue, a focused analysis will be needed to test specifically for the established symptoms of hypoglycemia. To this end, subjects will be studied for signs of tachycardia, anxiety, tremors, sweating, warmth, nausea or hunger (autonomic symptoms of hypoglycemia at [Glc] < 600 mg/l, i.e., <3.3 mM), and for fatigue, visual changes, confusion, dysarthria, dizziness, amnesia, lethargy, seizure or loss of consciousness (neuroglycopenic symptoms of hypoglycemia at [Glc] < 500 mg/l, i.e., <2.8 mM) [25,26].

A Se deficit may also exacerbate the course of hypoglycemia from any of the pathophysiological reasons mentioned above. In these conditions, supplemental Se may constitute a meaningful adjuvant treatment option, which needs to be tested thoroughly. Among the subjects at risk for Se deficiency are individuals residing in established Se-poor areas, those who are living exclusively on a restricted diet, patients with chronic disease, or those suffering from hepatic or gastrointestinal conditions causing a compromised Se uptake [27].

In light of our new findings, health issues that are considered to result from Se deficiency need to be re-evaluated for a potential contribution of Se-dependent hypoglycemia, e.g., elevated infection, autoimmune disease and cancer risks, or mortality upon major trauma [28,29], in critical disease [33] and notably, mortality from COVID-19 [31]. At present, it appears reasonable to test for Se deficiency in subjects presenting with hypoglycemia of unknown origin. In the future, this issue may become even more important as Se in soil and food is declining globally for geobiochemical reasons and climate change [32].

**Fig. 2:**
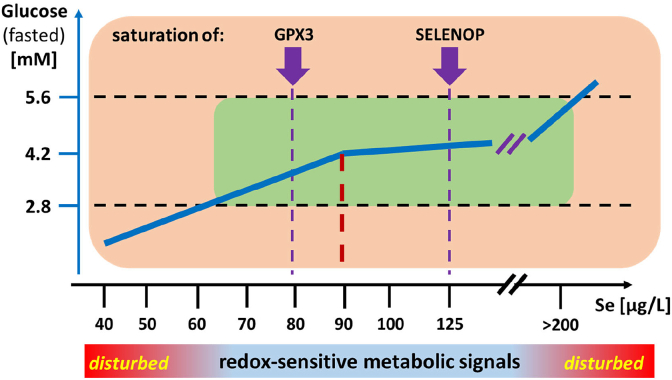
Hypothetical model on the interaction of Se status, redox-sensitive metabolic signaling and dysglycemia risk. The figure presents a potential relation of Se status (x-axis, serum Se) to euglycemia (fasted Glc between 2.8 and 5.6 mM). It is hypothesized that the observed relation in serum is related to selenoprotein expression, and intracellular redox-sensitive signaling of metabolic hormones including insulin that are amplified or suppressed in Se deficiency and excess, respectively. The model is compatible with Se-dependent selenoprotein expression in insulin target cells that is mirrored in the accessible data for the saturated expression of GPX3 in serum. Low Se status would cause e.g. a diminished GPX1 activity in insulin target cells, contributing to amplified insulin signals due to dysregulation of redox-regulated proteins like the insulin-antagonistic protein tyrosine phosphatase 1B (PTP1B). The inverse situation has been reported for elevated Se concentrations, up-regulating PTP1B and causing suppressed insulin signal ing. It remains to be studied in how far an optimized Se status (dark red line, at ca. 88.9 μg/l in serum) alleviates hypoglycaemia in Se-deficient subjects and patients, and protects from adverse health consequences of dysglycemia. (For interpretation of the references to colour in this figure legend, the reader is referred to the Web version of this article.)

A notable limitation of our study is the lack of functional data on glucose handling in Se deficiency, a focused search for symptoms of hypoglycemia in the Ningshan population, and additional mechanistic studies in model systems that would strengthen the hypothesized relation of insulin signaling, intracellular selenoprotein expression and glucose handling (Fig. 2). Among the strengths are the large group sizes, high degree of similarity of the populations studied and the strong and unambiguous relation of low Se with hypoglycemia.

## Support

National Key R&D Program of China (grant NO. 2018YFC1311500), and the 10.13039/501100001659Deutsche Forschungsgemeinschaft (10.13039/501100001659DFG Research Unit 2558 TraceAge, Scho 849/6-1 and TRR 296 LocoTact, Scho 849/7-1), Open Access Publication Fund of Charité–Berlin.

## 5 Contributions

YW, ER, QW, BS, and LS designed research; YW, ER, QW, HL, and CG conducted research; YW, ER, and QW analyzed data; YW, ER, BS, and LS wrote the paper; LS serves as guarantor, and had primary responsibility for final content. All authors read and approved the final manuscript.

## Declaration of competing interest

LS holds shares in selenOmed GmbH, a company involved in Se status assessment and supplementation. The other authors declare no conflict of interest in relation to this study.
